# Iron capture through CD71 drives perinatal and tumor-associated Treg expansion

**DOI:** 10.1172/jci.insight.167967

**Published:** 2024-07-02

**Authors:** Ilenia Pacella, Alessandra Pinzon Grimaldos, Alessandra Rossi, Gloria Tucci, Marta Zagaglioni, Elena Potenza, Valeria Pinna, Ivano Rotella, Ilenia Cammarata, Valeria Cancila, Beatrice Belmonte, Claudio Tripodo, Giuseppe Pietropaolo, Chiara Di Censo, Giuseppe Sciumè, Valerio Licursi, Giovanna Peruzzi, Ylenia Antonucci, Silvia Campello, Francesca Guerrieri, Valerio Iebba, Rita Prota, Maria Di Chiara, Gianluca Terrin, Valerio De Peppo, Gian Luca Grazi, Vincenzo Barnaba, Silvia Piconese

**Affiliations:** 1 Department of Translational and Precision Medicine, Sapienza University of Rome, Rome, Italy.; 2 Tumor Immunology Unit, Department of Health Sciences, University of Palermo, Palermo, Italy.; 3 Department of Molecular Medicine, Sapienza University of Rome, Rome, Italy.; 4 Laboratory affiliated to Istituto Pasteur Italia – Fondazione Cenci Bolognetti, Rome, Italy.; 5 Institute of Molecular Biology and Pathology (IBPM), National Research Council (CNR) of Italy, Sapienza University of Rome, Rome, Italy.; 6 Centre for Life Nano- & Neuro-Science, Fondazione Istituto Italiano di Tecnologia (IIT), Rome, Italy.; 7 Department of Biology, University of Rome Tor Vergata, Rome, Italy.; 8 Cancer Research Centre of Lyon (CRCL), UMR Inserm U1052/CNRS 5286, Lyon, France.; 9 Department of Medical, Surgical, and Health Sciences, University of Trieste, Trieste, Italy.; 10 Department of Maternal and Child Health, Sapienza University of Rome, Rome, Italy.; 11 Hepatobiliary and Pancreatic Surgery, IRCCS Regina Elena National Cancer Institute, Rome, Italy.; 12 Department of Internal Clinical Sciences, Anesthesiology and Cardiovascular Sciences, Sapienza University of Rome, Rome, Italy.; 13 Unità di Neuroimmunologia, IRCCS Fondazione Santa Lucia, Rome, Italy.

**Keywords:** Immunology, Metabolism, T cells, Tolerance

## Abstract

Besides suppressing immune responses, regulatory T cells (Tregs) maintain tissue homeostasis and control systemic metabolism. Whether iron is involved in Treg-mediated tolerance is completely unknown. Here, we showed that the transferrin receptor CD71 was upregulated on activated Tregs infiltrating human liver cancer. Mice with a Treg-restricted CD71 deficiency spontaneously developed a scurfy-like disease, caused by impaired perinatal Treg expansion. CD71-null Tregs displayed decreased proliferation and tissue-Treg signature loss. In perinatal life, CD71 deficiency in Tregs triggered hepatic iron overload response, characterized by increased hepcidin transcription and iron accumulation in macrophages. Lower bacterial diversity, and reduction of beneficial species, were detected in the fecal microbiota of CD71 conditional knockout neonates. Our findings indicate that CD71-mediated iron absorption is required for Treg perinatal expansion and is related to systemic iron homeostasis and bacterial gut colonization. Therefore, we hypothesize that Tregs establish nutritional tolerance through competition for iron during bacterial colonization after birth.

## Abstract 2

Iron uptake by Tregs is a critical requirement for Treg expansion that occurs physiologically post-birth and pathologically in tumors in both mice and men.

## Graphical abstract

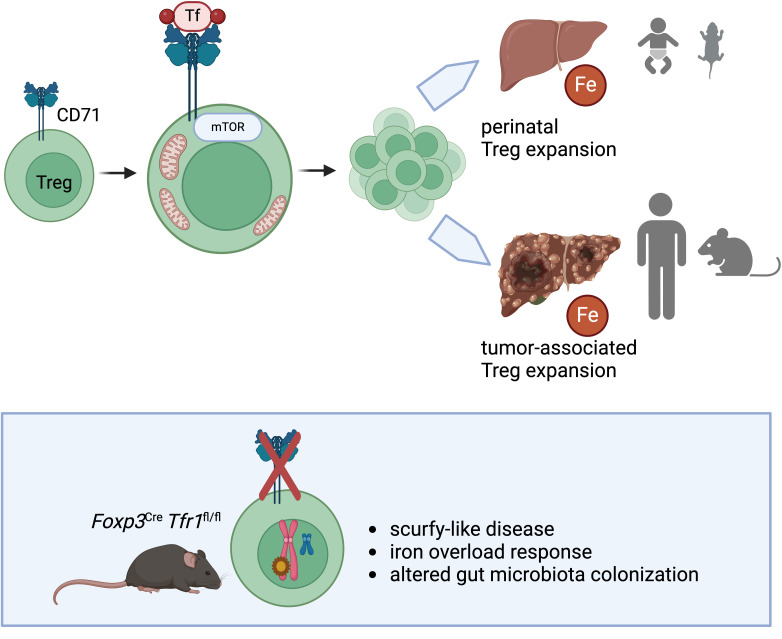

## Introduction

Regulatory T cells (Tregs) constitute a distinct subset of CD4^+^ T cells, expressing the lineage-specifying transcription factor Foxp3 and exerting nonredundant protection against spontaneous inflammation and autoimmunity. Mice and humans lacking Tregs develop an immune-mediated fatal multiorgan disorder known as scurfy or scurfy-like disease (1). The vital protective activity of Tregs is mediated not only by immune suppression but also by their multiple functions in tissue homeostasis, repair, and regeneration (2).

Tregs appear in the murine thymus 3 days after birth (3) and migrate to peripheral organs such as the liver, where they undergo considerable expansion in the perinatal life (4–7). Treg expansion has also been described in human neonates and in both mice and humans depends on microbial colonization (4, 5, 8). Perinatally expanded Tregs persist in adult mice (7), are responsible for the long-term maintenance of T cell anergy (6), and are enriched in the long-lived highly protective γREG^+^ subpopulation (9). In the first 2 weeks of life, lymphoid precursors colonize nonlymphoid organs, where tissue Tregs differentiate (2). In cancer, Tregs proliferate prominently and acquire a transcriptional program that blends tissue- and tumor-specific signatures (10). Treg proliferation requires a metabolic reprograming that involves increased nutrient internalization and degradation, macromolecule biosynthesis, and mitochondrial respiration (11). We have recently demonstrated that activated Tregs proliferated in cancer owing to a glycolytic-lipogenic metabolism (12, 13).

The field of immunometabolism is expanding in several directions, including “immunometallomics,” since metals like iron shape immune cell metabolism and functions (14). The control of systemic iron homeostasis by the hepatic hormone hepcidin represents a vital defense from infections, based on the competition for iron between host cells and microbes, a mechanism known as “nutritional immunity” (15–17). In T cells, the transferrin receptor 1 (CD71) is induced upon antigen encounter in an mTOR-dependent fashion (18) and supports IL-2 signal and mitochondrial function (19). T cells require iron for the proper development of effector and memory responses (20) and differentiation of pathogenic T helper lineages (21, 22). The *TFRC* gene, coding for CD71, belongs to a tumor Treg signature common to several cancers, including hepatocellular carcinoma (HCC) (23). However, the role of CD71 in Treg homeostasis and tumor-associated expansion is unknown.

Here, we revealed that CD71 was highly expressed by activated and tumor-infiltrating human Tregs, promoting their proliferation. In a conditional knockout (cKO) mouse model, we uncovered that Treg-restricted CD71 loss compromised Treg perinatal expansion and tissue Treg differentiation and was associated with dysfunctions in systemic iron metabolism and nutritional immunity to bacteria.

## Results

### Activated human Tregs express the transferrin receptor in vitro and in HCC.

**Figure 1:**
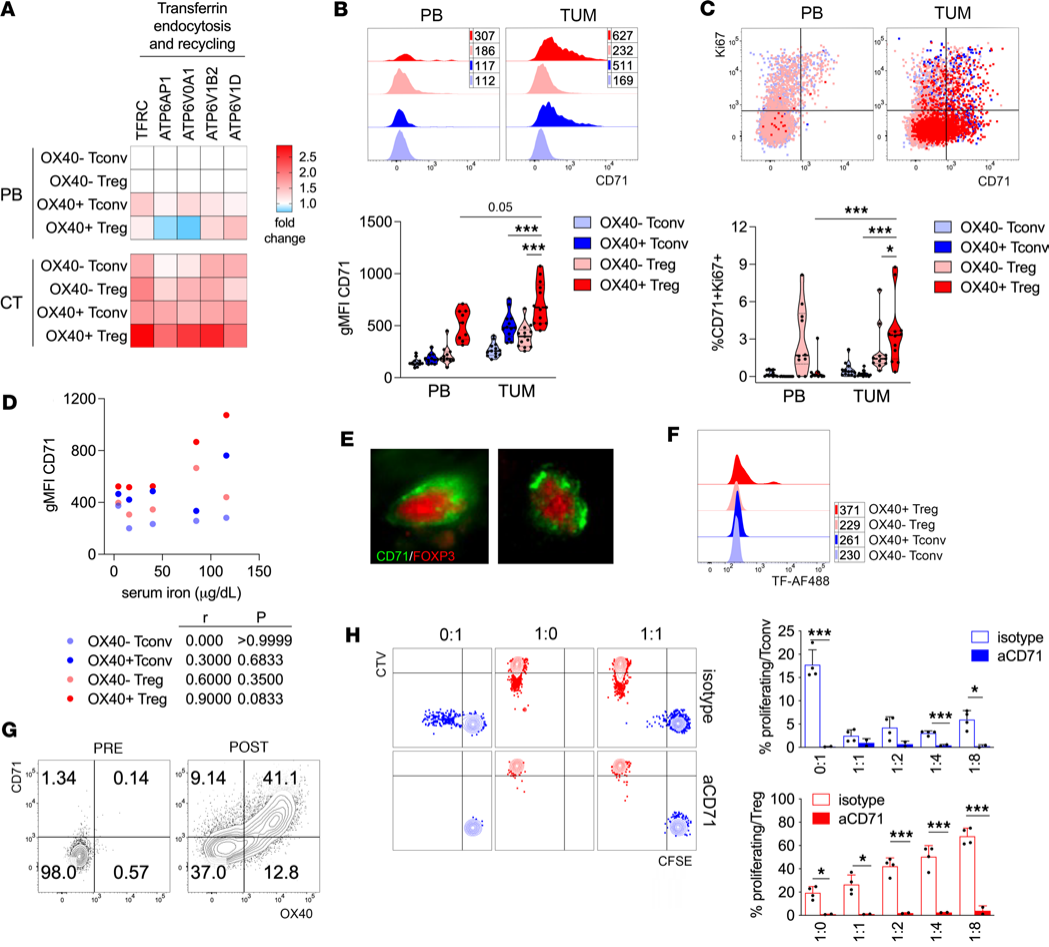
Human Tregs express CD71 when activated in vitro or in vivo in tumor. (**A**) OX40^+^/OX40^–^ CD45RA^lo^ Tregs and conventional T cells (Tconvs) were sorted from the peripheral blood (PB) or cirrhosis/tumor (CT) of 5 patients with HCC, and gene expression analysis was performed (12). The heatmap displays the fold-change over the respective control (OX40^–^ Tconvs or Tregs in PB) in the genes accounting for the pathway of interest, showing statistically significant enrichment (Reactome, *P* < 0.05). (**B** and **C**) Representative plots (upper panel) and cumulative data (lower panel) showing CD71 expression (**B**) or CD71^+^Ki67^+^ percentage (**C**) in the indicated cell subsets (CD45RA^lo^) from matched samples of PB or tumor (TUM) from patients with HCC (*n* = 13). Numbers in the histogram plot indicate the geometric mean fluorescence intensity (gMFI) of CD71. *P < 0.05, ***P < 0.005, by Wilcoxon’s matched pairs signed rank test. (**D**) Spearman’s correlation between CD71 expression in the indicated cell subsets from TUM samples, and serum iron concentrations, in patients with HCC (*n* = 5). (**E**) Immunofluorescence of CD71 and FOXP3 in a representative HCC sample. Original magnification, 63×. (**F**) Fluorescent transferrin internalization in the indicated cell subsets from representative HCC sample. Numbers indicate the gMFI. (**G**) Expression of CD71 and OX40 on Tregs from PB of a representative healthy donor (HD), either freshly isolated (PRE) or after 2 weeks’ expansion in vitro (POST). (**H**) In vitro–expanded and CellTrace Violet–labeled (CTV-labeled) Tregs (shown in red) were tested in standard suppression assay at different ratios against autologous CFSE-labeled Tconvs (blue), plus anti-CD71 blocking mAb or isotype control. Representative profiles of dye dilution (left panel) and cumulative analysis of proliferating cell percentages (right panel) are shown. Ratios indicate the Treg/Tconv proportions. Data shown are from a representative HD out of 2. Each condition was tested in 2–4 replicates. Bars indicate means ± SD. *P < 0.05, ***P < 0.005, by Student’s *t* test, unpaired.

A pathway enrichment analysis of our gene expression profiling of OX40^+^ Tregs, from human liver cirrhosis/tumor (CT) samples (12), uncovered transferrin endocytosis and recycling among pathways specifically upregulated in CT OX40^+^ Tregs. The genes for CD71 (*TFRC*) and for vacuolar ATPases, involved in endosomal acidification and CD71 recycling, were included in this pathway (Figure 1A). We validated this result through flow cytometry in an independent cohort of patients with HCC (Supplemental Table 1; supplemental material available online with this article; https://doi.org/10.1172/jci.insight.167967DS1): CD71 expression in tumor-infiltrating OX40^+^ Tregs was significantly higher than OX40^+^ Tconvs or OX40^–^ Tregs and tended to be higher also than OX40^+^ Tregs in peripheral blood (PB), where they were represented at very low frequency (12, 13) (Figure 1B). CD71 expression and proliferation were associated in intratumor OX40^+^ Tregs: indeed, the percentage of CD71^+^Ki67^+^ cells was significantly higher in OX40^+^ Tregs in tumor, with respect to controls (Figure 1C). A positive association trend (*P* = 0.0833) between serum iron and CD71 expression was found for tumor OX40^+^ Tregs only (Figure 1D), suggesting that circulating iron availability may affect the intratumor pool of CD71^+^ Tregs. Immunofluorescence analysis validated CD71 expression by HCC-infiltrating FOXP3^+^ cells and unveiled CD71 clustering on the cell surface (Figure 1E). In line with their higher CD71 expression, tumor-infiltrating OX40^+^ Tregs displayed a stronger capacity to internalize a fluorescently labeled transferrin ex vivo (Figure 1F).

To study the consequences of CD71 blockade on Treg functions, we used an in vitro protocol of Treg expansion, where a significant Treg proportion coexpressed OX40 and CD71 (Figure 1G), similarly to what we observed ex vivo from HCC. Then, Tregs were stimulated for a further 7 days, either alone or with increasing ratios of autologous Tconvs: the addition of an anti-CD71 blocking antibody profoundly inhibited the proliferation not only of Tconvs, as expected, but also of Tregs, but did not revert Treg suppressive function (Figure 1H). Despite the limitation of this experiment (which does not allow discriminating between Tconv resistance to activation and susceptibility to suppression, under CD71 blockade), these data demonstrate a key role for CD71 in the in vitro proliferation of human activated Tregs.

### Mice with Treg-restricted CD71 deficiency develop a scurfy-like disease.

**Figure 2:**
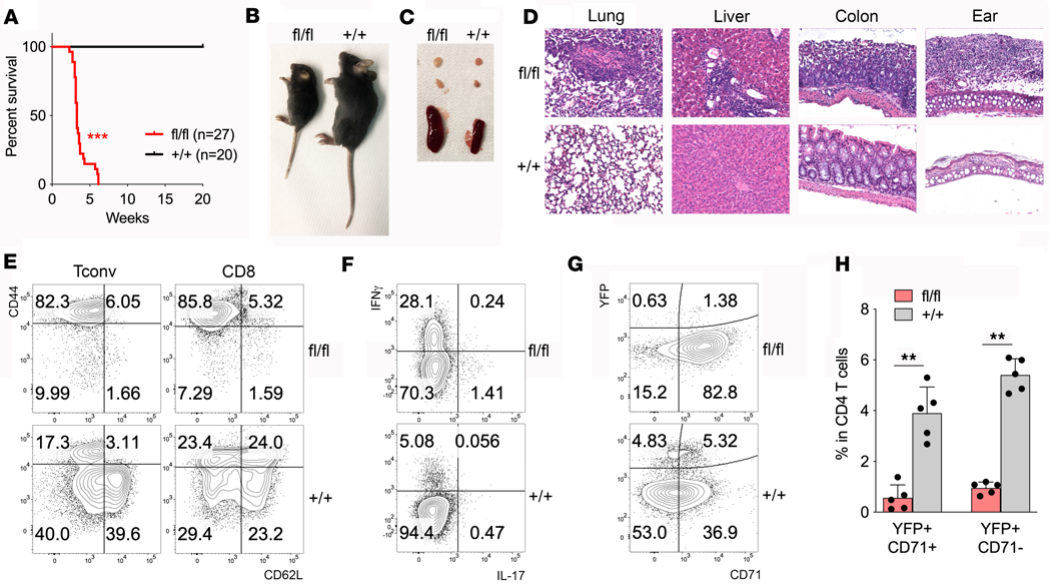
Treg-restricted CD71 deficiency induces a scurfy-like disease. (**A**) Survival curve of *Foxp3*^Cre^ *Tfrc*^fl/fl^ mice (fl/fl, red), compared with *Foxp3*^Cre^ *Tfrc*^+/+^ littermates (+/+, black). ***P < 0.005. (**B** and **C**) Pictures of representative mice (**B**) and spleen and inguinal lymph nodes (**C**) for each genotype, at 3–4 weeks of age. (**D**) Hematoxylin-eosin staining from 1 mouse out of 3 for each genotype. Original magnification, 20×. (**E**) CD44/CD62L expression in gated Tconvs (CD4^+^YFP^–^) or CD8^+^ T cells in a representative spleen. (**F**) IFN-γ and IL-17 production by Tconvs (CD4^+^YFP^–^) in a representative spleen. (**G** and **H**) Representative contour plots (**G**) and cumulative analysis (**H**) of CD71^+^ and CD71^–^ YFP^+^ percentages in the spleen (*n* = 5/group). Bars indicate means ± SD. **P < 0.01, by Mann-Whitney *U* test.

We generated a cKO mouse model constitutively deficient of CD71, selectively in Tregs. To this aim, *Foxp3*^Cre/Cre^ mice were crossed with *Tfrc*^fl/fl^ mice to obtain a progeny of *Foxp3*^Cre^ (comprising both *Foxp3*^Cre/Y^ hemizygous males and *Foxp3*^Cre/Cre^ homozygous females) *Tfrc*^fl/fl^ cKO mice, compared with *Foxp3*^Cre^ *Tfrc*^+/+^ littermates as control. The frequencies of all the genotypes in the progeny were in line with mendelian inheritance (not shown). However, we noticed that cKO mice spontaneously developed a lethal disorder, leading to death at 3–6 weeks of age (Figure 2A), and characterized by severe growth reduction, scaly skin (Figure 2B), splenomegaly, and lymphadenopathy (Figure 2C). At microscopic inspection at sacrifice (at 3–4 weeks of age), all the analyzed organs (lung, liver, colon, and ear) displayed severe inflammation and subverted architecture (Figure 2D and Supplemental Figure 1B). The *Foxp3*^Cre^ *Tfrc*^+/fl^ heterozygous progeny did not display reduced survival or macroscopic defect (not shown), even though a slightly increased organ inflammation was observed (Supplemental Figure 1B). In lymphoid organs of cKO mice, most CD4^+^ Tconvs and CD8^+^ T cells presented a CD44^+^CD62L^–^ effector memory phenotype (Figure 2E), and both Th1 and Th17 cells were expanded (Figure 2F). At an earlier age, Tconvs from cKO mice produced significantly more IFN-γ as well as type 2 cytokines (IL-4, IL-5, IL-13) (Supplemental Figure 1A). These data indicate that the spontaneous disorder in cKO mice has all the features of a scurfy-like disease (24), and that the sole Treg-restricted CD71 ablation was sufficient to phenocopy the complete Foxp3 deficiency.

We then asked whether the scurfy-like disease developed because of reduced Treg frequencies (especially CD71^+^) or impaired Treg functions. In 3- to 4-week-old control mice, around half of YFP^+^ Tregs expressed CD71 (Figure 2G). In cKO mice, a strong and statistically significant reduction was observed not only in CD71^+^ but also CD71^–^ Tregs (Figure 2, G and H). Concomitantly, the YFP^–^ compartment, representing Tconvs, strongly upregulated CD71 (Figure 2G), indicative of their activation. Treg reduction occurred not only in lymphoid organs but also in the colonic lamina propria (LP) (Supplemental Figure 1C). Intestinal Tregs exist in 2 subsets, identified by Helios or RORγt (25): only a minority of RORγt^+^ Tregs was preserved in cKO mice (Supplemental Figure 1, D and E).

To explore the impact of Treg-specific CD71 haploinsufficiency on tumor growth, we exploited the *Foxp3*^Cre^ *Tfrc*^+/fl^ progeny: in their lymphoid organs, there was a trend for reduced frequency of CD71^+^ Tregs, which was compensated by CD71^–^ Tregs (Supplemental Figure 2, A and B), leading to a comparable frequency of total Tregs (Supplemental Figure 2C), expressing significantly less CD71 (Supplemental Figure 2D). When the HCC line 18.5 (26) was subcutaneously injected, no difference in tumor growth (Supplemental Figure 2E) or Treg expansion (Supplemental Figure 2, F and G) was observed. Of interest, the difference in CD71 expression by Tregs, maintained in lymph nodes, was lost in the tumor, where CD71 was significantly upregulated by Tregs in both groups (Supplemental Figure 2, H and I). This result demonstrates that CD71 haploinsufficiency in Tregs is compensated at the tumor site and strengthens the notion that CD71 plays pivotal roles in tumor-Treg expansion.

### CD71 is involved in Treg perinatal expansion in both mice and humans.

**Figure 3:**
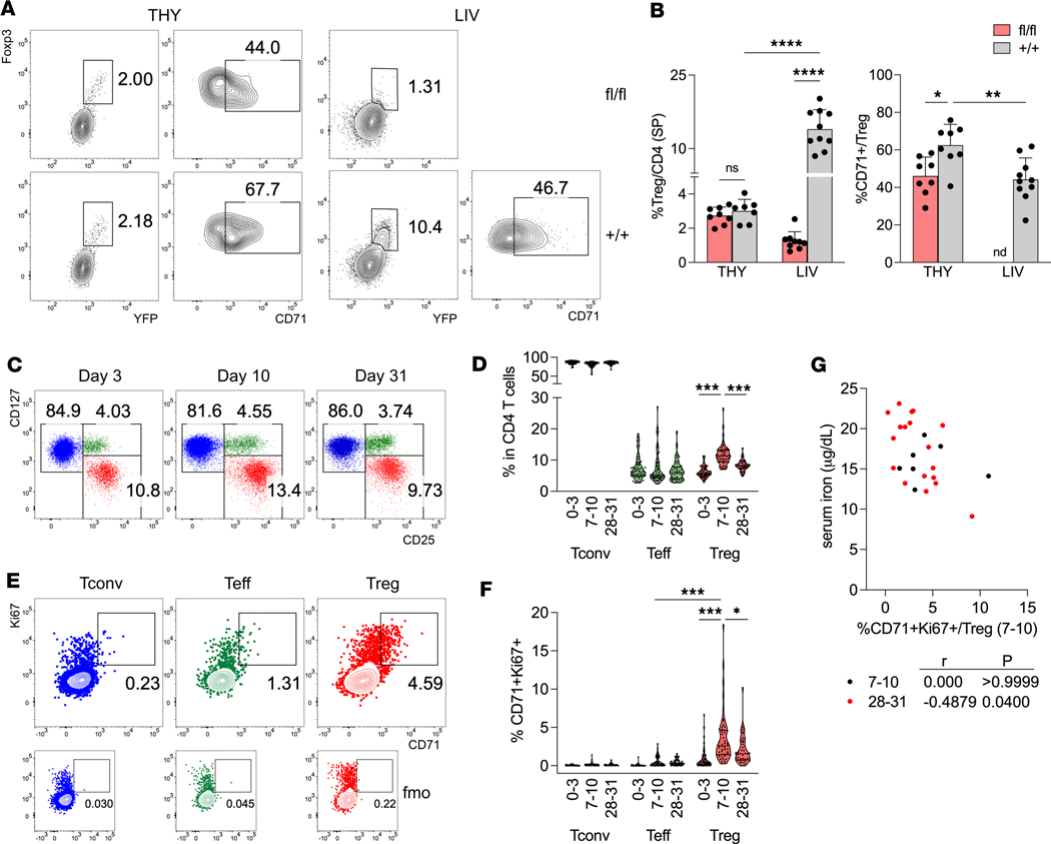
CD71 is involved in Treg perinatal expansion in both mice and humans. (**A** and **B**) Representative contour plots (**A**) and cumulative analysis (**B**) showing Treg (YFP^+^Foxp3^+^) percentages, and CD71^+^ percentages in gated Tregs, among TCRβ^+^CD3^+^CD4^+^CD8^–^ single-positive (SP) cells from thymus (THY), or among TCRβ^+^CD3^+^CD4^+^ T cells from liver (LIV), of *Foxp3*^Cre/Y^ *Tfrc*^fl/fl^ male mice (fl/fl, red), compared with *Foxp3*^Cre/Y^ *Tfrc*^+/+^ male littermates (+/+, gray), aged 8–10 days. Data are from 7–10 samples/group, pooled from 2 independent experiments. Bars indicate means ± SD. *P < 0.05, **P < 0.01, ****P < 0.0001, by ordinary 2-way ANOVA with Tukey’s multiple comparisons test. nd, not determined. (**C** and **D**) Representative plots (**C**) and cumulative analysis (**D**) of percentages of Tconvs (CD127^hi^CD25^lo^), Teffs (CD127^hi^CD25^int^), and Tregs (CD127^lo^CD25^hi^) from PB of human preterm neonates (*n* = 53), collected at 0–3, 7–10, or 28–31 days after birth. (**E** and **F**) Representative plots (**E**) and cumulative analysis (**F**) of CD71^+^Ki67^+^ cell percentages in gated Tconvs (blue), Teffs (green), and Tregs (red), in a representative sample at day 10. Fluorescence minus one (FMO) control in each gate is shown. *P < 0.05, ***P < 0.005, by Wilcoxon’s matched pairs signed rank test. (**G**) Spearman’s correlation between CD71^+^Ki67^+^ frequency in Tregs, and serum iron concentrations in neonates, at 7–10 (*n* = 7) or 28–31 (*n* = 18) days after birth.

The above results suggested that CD71 deficiency compromised Treg development at very early ages, when a higher proportion of Tregs transiently expressed CD71. Considering that CD71 is highly represented in thymocytes and dictates their development (27, 28), and aiming to discriminate between defective thymic development and reduced perinatal expansion in cKO mice, Treg frequency and CD71 expression were estimated in thymuses and livers at 8–10 days after birth, when perinatal Treg expansion peaks in the liver (4–6). In CD4^+^CD8^–^ single-positive thymocytes of cKO mice, a comparable Treg frequency, but a significantly lower CD71 expression, was detected (Figure 3, A and B), possibly denoting incomplete Cre-mediated excision of the *Tfrc* gene at this stage. In the livers of control mice, Tregs were represented at high percentages, as expected, and contained fewer CD71^+^ cells than the thymus, validating the idea that most Tregs had expressed CD71 transiently during their thymic development (Figure 3, A and B). Virtually no Tregs were found in the livers of cKO mice (Figure 3, A and B), while Tconvs displayed a highly activated (CD44^hi^CD71^+^PD1^+^) phenotype (Supplemental Figure 3). Together these data indicated that defective perinatal expansion, rather than impaired thymic development, accounted for the profound Treg reduction in cKO mice.

Perinatal Treg expansion also occurs in human preterm neonates, peaking 7–8 days after birth (8). In a cohort of 53 preterm neonates (Supplemental Table 2), we validated that Treg percentage significantly increased from days 0–3 to 7–10 after birth and returned to almost basal levels after 28–31 days (Figure 3, C and D). A tendency for higher Treg frequency at days 7–10 was found in infants born from mothers not receiving antenatal antibiotics, as previously published (8), or receiving antenatal iron supplementation (not shown). Of note, at the Treg peak at days 7–10, the frequency of proliferating (Ki67^+^) Tregs coexpressing CD71 was significantly increased, compared with other time points or with T effector (Teff; CD127^+^CD25^+^) cells (Figure 3, E and F): this observation indicates that CD71 upregulation associates with perinatal Treg expansion in humans. The percentage of CD71^+^Ki67^+^ Tregs at 7–10 days significantly and negatively correlated with serum iron concentration at 28–31, not at 7–10, days (Figure 3G), suggesting that an early CD71^+^ Treg proliferation may result in reduced circulating iron at subsequent time points.

### CD71 dictates Treg mitochondrial fitness and tissue Treg development.

**Figure 4:**
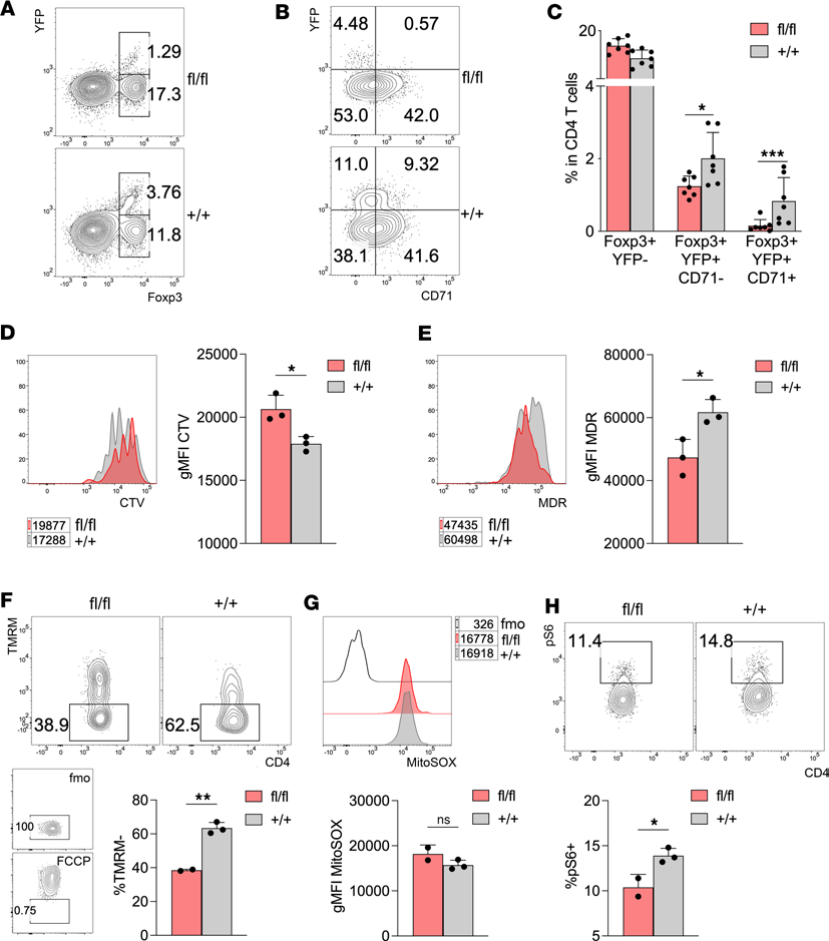
CD71 sustains Treg metabolic fitness in a cell-intrinsic fashion in healthy mosaic females. (**A**–**C**) Representative contour plots (**A** and **B**) and cumulative analysis (**C**) of YFP^–^ and YFP^+^ Foxp3^+^ percentages in gated CD4^+^ T cells (**A**), and CD71^+^ YFP^–^ and YFP^+^ percentages in gated Foxp3^+^ (**B**), from the spleens of *Foxp3*^Cre/+^ *Tfrc*^fl/fl^ (fl/fl, red) and *Foxp3*^Cre/+^ *Tfrc*^+/+^ (+/+, gray) female littermates, aged 8–12 weeks. Data are from 7 samples/group, pooled from 2 independent experiments. Bars indicate means ± SD. *P < 0.05, ***P < 0.005, by Mann-Whitney test. (**D** and **E**) CTV dilution (**D**) and MDR staining (**E**) in gated YFP^+^ from CD4^+^ T cells isolated from the spleens of *Foxp3*^Cre/+^ *Tfrc*^fl/fl^ or *Tfrc*^+/+^ females and polyclonally stimulated in vitro for 3–4 days. Numbers indicate the gMFI. Data shown are from 1 representative out of 2 independent experiments. Each condition was tested in triplicates. Bars indicate means ± SD. *P < 0.05, by Student’s *t* test, unpaired. (**F**–**H**) CD4^+^YFP^+^ cells were sorted from spleens of *Foxp3*^Cre/+^ *Tfrc*^fl/fl^ (fl/fl) or *Foxp3*^Cre/+^ *Tfrc*^+/+^ (+/+) female littermates of 8–12 weeks of age and cultured in vitro for 18 hours with coated anti-CD3 and IL-2. (**F**) TMRM staining, performed in quenching mode. The percentages of TMRM^–^ cells, corresponding to cells with hyperpolarized mitochondria, are shown. Insets display negative (FMO) and positive (FCCP-treated) controls. (**G**) MitoSOX staining, expressed as gMFI. (**H**) Frequency of pS6^+^ cells. Data are from 1 experiment representative of 2. Each condition was tested in 2–3 replicates. *P < 0.05, **P < 0.01, by unpaired Student’s *t* test.

To identify the direct mechanisms restraining Treg expansion in the absence of CD71, we could not perform further analyses in cKO mice, which harbored extremely low Treg frequencies, not allowing their isolation and characterization. Moreover, their strong systemic inflammation might affect Treg function indirectly, in a CD71-independent fashion. Therefore, we took advantage of *Foxp3*^Cre/+^ *Tfrc*^fl/fl^ mosaic female mice, where roughly half of Tregs express Cre-YFP according to the random X chromosome inactivation (29). These mice were not affected by any macroscopic sign of scurfy-like disease (not shown) and were thus suitable to study Treg-intrinsic and inflammation-independent events driven by CD71 deficiency. In line with other reports (30, 31), less than half of Foxp3^+^ cells coexpressed YFP in *Foxp3*^Cre/+^ *Tfrc*^+/+^ controls, but this frequency significantly dropped in *Foxp3*^Cre/+^ *Tfrc*^fl/fl^ mice, while a compensatory Foxp3^+^YFP^–^ population increased (Figure 4A). Mirroring what observed in cKO mice, both CD71^+^ and CD71^–^ Tregs were reduced in the YFP^+^ compartment of *Foxp3*^Cre/+^ *Tfrc*^fl/fl^ mice compared with controls (Figure 4, B and C), corroborating the notion that CD71 deficiency affected the whole Treg compartment in a cell-intrinsic fashion.

When CD4^+^ T cells from *Foxp3*^Cre/+^ *Tfrc*^fl/fl^ mice were polyclonally stimulated ex vivo, the YFP^+^ compartment proliferated less compared with control cells (Figure 4D). CD71-deficient Tregs, sorted from *Foxp3*^Cre/+^ *Tfrc*^fl/fl^ mice, not only proliferated less but also displayed lower survival among the dividing cells (Supplemental Figure 4). This event was associated with a reduced polarized mitochondrial mass, as detected with the MitoTracker Deep Red (MDR) dye (Figure 4E), and with a decreased frequency of cells with hyperpolarized mitochondria, as revealed by the tetramethylrhodamine methyl ester (TMRM) dye (Figure 4F). However, the production of mitochondrial superoxide was not affected by CD71 deficiency (Figure 4G). In line with the notion that CD71 drives mTOR activation (18), we observed lower pS6^+^ frequency among CD71-deficient Tregs (Figure 4H).

**Figure 5:**
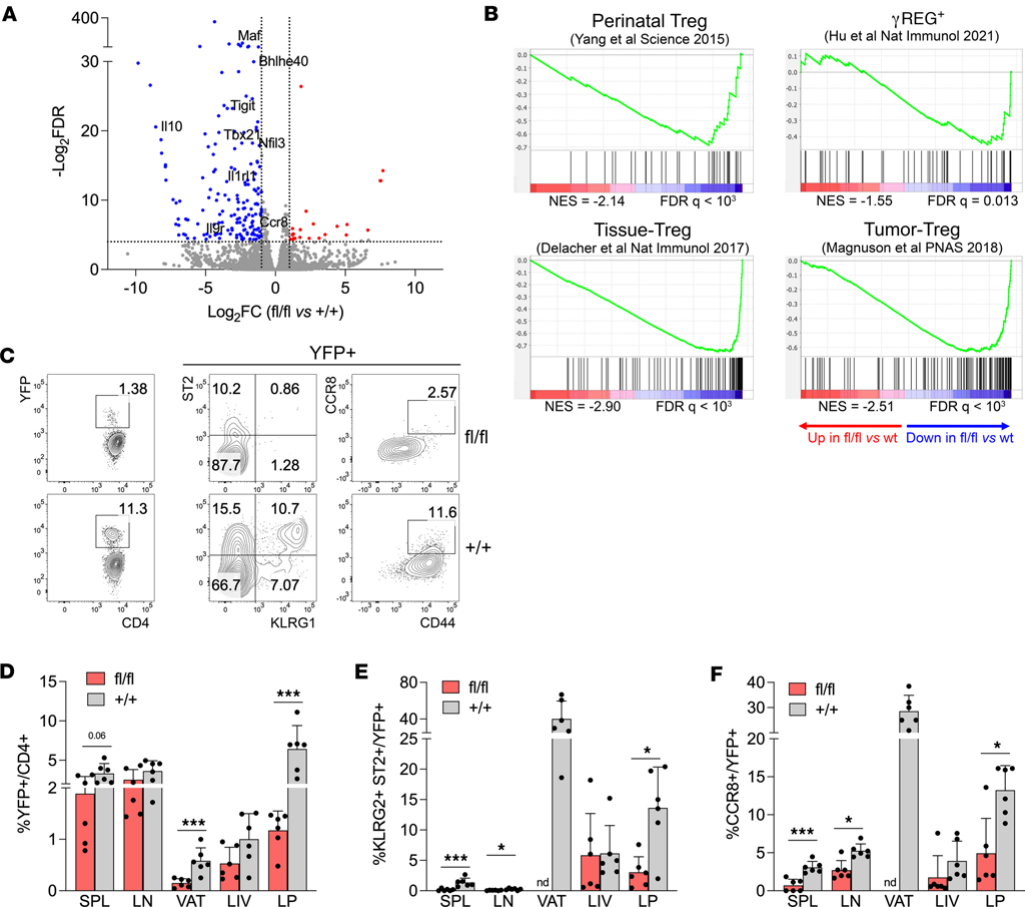
CD71 deficiency compromises tissue Treg development. (**A**) Volcano plot showing the fold-change in gene expression by RNA-Seq of CD4^+^YFP^+^ cells sorted from spleens (*n* = 3/group) of *Foxp3*^Cre/+^ *Tfrc*^fl/fl^ (fl/fl) and *Foxp3*^Cre/+^ *Tfrc*^+/+^ (+/+) female littermates, aged 8–12 weeks. Genes with FDR < 0.05 and with fold-change > 2 (red) or <–2 (blue) in fl/fl versus +/+ are highlighted. (**B**) Gene set enrichment analysis of the transcriptome of *Foxp3*^Cre/+^ *Tfrc*^fl/fl^ versus *Tfrc*^+/+^ Tregs. Gene sets were obtained from published signatures of perinatal Tregs (7), γREG^+^ (9), tissue Tregs (32), or tumor Tregs (33). Normalized enrichment scores (NES) and FDR *q* values are shown under each plot. (**C**) Representative plots of the analysis of the colonic LP of *Foxp3*^Cre/+^ *Tfrc*^fl/fl^ and *Tfrc*^+/+^ female mice, showing YFP^+^ in gated CD4^+^ T cells (left panels) or KLRG1^+^ST2^+^ (middle panels) or CD44^+^CCR8^+^ (right panels) tissue Tregs in gated YFP^+^ cells. (**D**–**F**) Percentages of YFP^+^ cells (**D**) or KLRG1^+^ST2^+^ (**E**) or CD44^+^CCR8^+^ (**F**) tissue Tregs in gated YFP^+^ cells, in spleen (SPL), inguinal lymph node (LN), VAT, liver (LIV), and colonic LP. Data are from 6 samples/group, pooled from 2 independent experiments. Bars indicate means ± SD. *P < 0.05, ***P < 0.005, by Mann-Whitney test. nd, not determined.

A transcriptomic analysis of YFP^+^ Tregs sorted from *Foxp3*^Cre/+^ *Tfrc*^fl/fl^ and *Tfrc*^+/+^ mice showed that the genes significantly downregulated in CD71-deficient Tregs greatly outnumbered the upregulated ones (24 and 163 DEGs with FDR < 0.05 and fold-change >2 or <–2, respectively) and included genes with well-known roles in Treg expansion, differentiation, migration, and suppressive function, such as *Maf*, *Il10*, *Tbx21*, *Nfil3*, *Bhlh40* (encoding DEC1), and *Ccr8* (Figure 5A). The lower protein content of DEC1, NFIL3, and c-MAF was verified through flow cytometry (Supplemental Figure 5). The gene set enrichment analysis (GSEA) revealed that CD71-deficient Tregs had significant reduction of 4 signatures related to perinatal expansion (7), γREG^+^ (9), tissue Tregs (32), or tumor Tregs (33) (Figure 5B).

To validate the defective tissue Treg development, we analyzed, in several tissues of *Foxp3*^Cre/+^ *Tfrc*^fl/fl^ healthy mosaic females, the frequency of total YFP^+^ Tregs and, among these, the proportion of tissue Tregs, identified as KLRG1^+^ST2^+^ (32, 34), or CCR8^+^ cells (2) (Figure 5C). YFP^+^ Treg percentage was reduced in visceral adipose tissue (VAT), colonic lamina propria (LP), and (not significantly) liver, to a higher extent than lymphoid organs (Figure 5, C and D), denoting a stronger reliance of tissue Tregs on CD71. Tissue Tregs, abundant among YFP^+^ Tregs in both VAT and LP of control mice, were profoundly reduced in *Foxp3*^Cre/+^ *Tfrc*^fl/fl^ mice (Figure 5, E and F), which demonstrated a CD71 requirement for proper tissue Treg development.

### CD71 loss in Tregs associates with dysregulated iron systemic metabolism and gut microbial colonization.

Immune cells, including Tregs, exert their host-protective activities by collaborating with epithelia and commensal microbiota, often through metabolites in early life (35). Since Treg perinatal expansion is related to microbial colonization (5, 8), and since iron is a vital element for bacteria, dictating the balance between pathogens and commensals (16), we hypothesized that the CD71-dependent perinatal Treg proliferation provoked iron consumption, thus impacting systemic iron homeostasis and consequently gut microbial colonization. Supporting this idea, the labile iron content in Tregs, measured with Calcein Blue AM, was higher in liver and even more in the colon of neonate mice than spleen of adult mice (Supplemental Figure 6).

**Figure 6:**
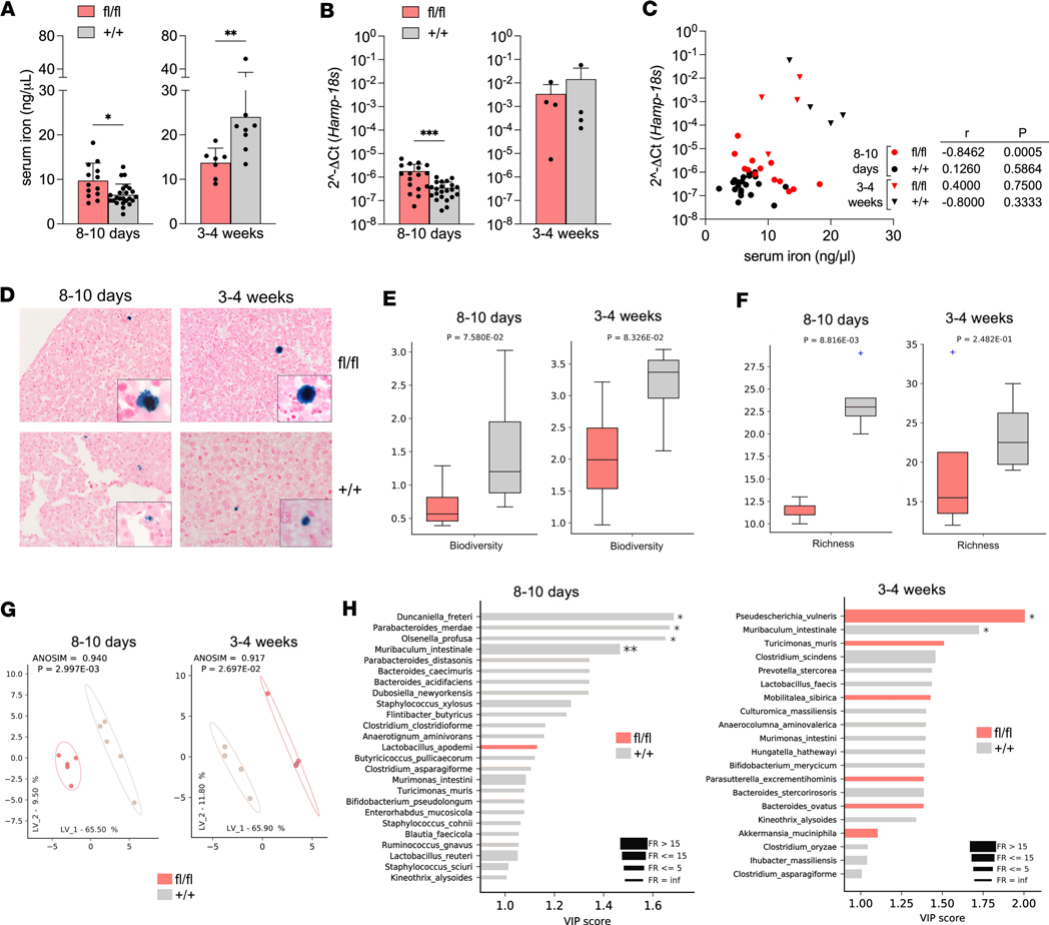
Treg-intrinsic CD71 deficiency affects systemic iron status and gut microbial colonization. (**A**) Systemic iron concentration in the serum of *Foxp3*^Cre/Y^ *Tfrc*^fl/fl^ (fl/fl, red), and *Foxp3*^Cre/Y^ *Tfrc*^+/+^ (+/+, gray) male littermates, at 8–10 days or 3–4 weeks of age. (**B**) *Hamp* gene expression by real-time reverse transcription PCR (RT-PCR) in whole liver extracts. Data are from 4–24 samples/group, pooled from 3 independent experiments. Bars indicate means ± SD. *P < 0.05, **P < 0.01, ***P < 0.005, by Mann-Whitney test. (**C**) Spearman’s correlation between serum iron and hepatic *Hamp* expression. (**D**) Perls Prussian blue staining in liver specimens of *Foxp3*^Cre/Y^ *Tfrc*^fl/fl^ or *Tfrc*^+/+^ males at 8–10 days or 3–4 weeks of age. Representative images of 2–4 mice/group are shown. Original magnification, 10×. Insets, 40×. (**E**–**G**) α Diversity measurements of biodiversity (Shannon, **E**) and richness (observed operational taxonomic units, **F**) and partial least-squares discriminant analysis (PLS-DA) (**G**), of the microbial species identified through 16s rRNA gene sequencing of DNA extracted from stools of *Foxp3*^Cre/Y^ *Tfrc*^fl/fl^ (red) or *Tfrc*^+/+^ (gray) males at 8–10 days or 3–4 weeks of age (*n* = 4–5 mice/group). Box plots show the interquartile range, median (line), and minimum and maximum (whiskers). (**H**) Variable importance plot (VIP) describing the most discriminant species in descending order of importance. Each bar reports the following information: (i) length, VIP score; (ii) bar color, cohort in which the species has the highest average relative abundance (high); (iii) thickness, fold ratio (FR) among the 2 groups considered; (iv) significance of Mann-Whitney *U* test between high and low (*P < 0.05, **P < 0.01).

A significantly higher iron concentration was found in the sera of 8- to 10-day-old cKO mice, at the peak of perinatal expansion; conversely, hypoferremia appeared at 3–4 weeks of age, probably in response to systemic inflammation (Figure 6A). The hepatic expression of hepcidin (induced by iron overload and inhibiting iron efflux; ref. 16) was indeed significantly higher in cKO mice at 8–10 days (Figure 6B) and inversely correlated with iron concentrations (Figure 6C). Of note, it did not correlate with the serum concentration of IL-6 as a marker of systemic inflammation, even though IL-6 was already significantly increased in cKO mice at that age (Supplemental Figure 7). The iron stores were enlarged in intrahepatic macrophages in cKO mice, as revealed through Perls staining (Figure 6D). These results indicate that CD71 deficiency in Tregs associates with the hepatic iron overload response at an early time point.

Finally, we analyzed, through 16s rRNA gene sequencing, the fecal microbiota diversity and composition at 8–10 days or 3–4 weeks after birth in cKO mice and control littermates. At both time points, 2 indices, Shannon biodiversity (Figure 6E) and richness (Figure 6F), were significantly lower in cKO mice. In a supervised ordination plot, the 2 sample groups segregated in significantly distant clusters (Figure 6G). At 3–4 weeks of age, *P*. *vulneris* was relatively enriched in feces of cKO mice (Figure 6H): this microbe is a Gammaproteobacterium, a class enriched in inflammatory bowel disease (36). Conversely, at 8–10 days of age, several species were relatively reduced in cKO mice: these included *D*. *freteri*, *P*. *merdae,* *M*. *intestinale* (all Bacteroidetes), and *O*. *profusa* (an Actinobacterium) (Figure 6H). These 2 phyla comprise several species with beneficial effects for immunity, which decrease in gut inflammation (36). In summary, CD71 deficiency in Tregs correlates not only to an iron overload response but also to a reduced diversity of gut microbial species and a decrease of protective strains. These events occurred in cKO neonates already at 8–10 days after birth, coinciding with the peak of perinatal Treg expansion, not correlating with serum IL-6 concentration, and before overt inflammation.

## Discussion

Beyond their well-recognized role as immunosuppressive cells, Tregs modulate tissue and systemic metabolism: indeed, Treg defects were shown to affect tissue programs related to lipid metabolism in the liver (37) or to glucose metabolism in the VAT (38). Here, we uncovered that Treg proliferation required iron capture through CD71 and that its failure was associated with an iron overload response and a dysregulated gut microbiota colonization.

In line with previous transcriptomic data (23), HCC-infiltrating human Tregs expressed CD71 and internalized transferrin more than Tconvs, in association with proliferation and OX40 expression, which labels stable and activated Tregs (12, 13). CD71 was coinduced with OX40 also in Tregs activated in vitro, suggesting that CD71 may not be inherent to the tumor Treg signature but may rather mark metabolically activated Tregs enriched in tumors. Accordingly, CD71 was upregulated on cycling Tregs in human and murine neonates. Others have described that CD71 counteracts induced Treg (iTreg) polarization (21), similarly to several other contexts of nutrient deprivation (11). While iron restriction may promote iTregs in pathological settings, iron availability may be required for the physiological expansion of thymic Tregs in the early life.

Whether microenvironmental and circulating iron availability impacts Treg expansion is unknown. In a small cohort of patients with HCC, CD71 expression on tumor Tregs positively correlated with serum iron concentrations; in human neonates, Treg perinatal expansion tended to be higher in neonates from mothers receiving iron supplementation antenatally. These findings suggest that iron provision may foster immune regulation in contexts that are physiologically or pathologically characterized by a Treg advantage, which are perinatal life and tumor growth, respectively. In the former case, a poor iron status during pregnancy is associated with increased risk of asthma and atopic outcomes in children (39): whether this involves Treg defects has not been investigated. In the case of tumor, cancer cells are “iron-addicted” (40), thus depriving T cells of this nutrient, among others. Our data indicate that CD71^+^ Tregs may act in similar fashion in the tumor microenvironment and that iron supplements should be considered with caution in patients with cancer, since they could fuel tumor Treg expansion.

We generated a mouse model of Treg-specific CD71 deficiency that spontaneously developed a scurfy-like disease, caused by a virtually complete absence of perinatal Treg expansion, which compromised the development of tissue Tregs in several districts; notably, the tissue Treg signature (including Nfil3) was reduced in CD71-null Tregs sorted from spleens, thus suggesting that CD71 deficiency might affect the development of the lymphoid Nfil3^+^ precursor of tissue Tregs (34). It is conceivable that tissue Treg precursor differentiation requires proliferation: both events occur in the perinatal life (2) and in secondary lymphoid organs (6), and splenic Nfil3^+^ Tregs are highly proliferative (34). From our model, we cannot exclude that CD71 did play a role also in Treg development in the thymus, since the *Tfrc* gene was not completely deleted, and CD71 protein still partially maintained, in Foxp3^+^ thymocytes. However, the decline in CD71-deficient Tregs was massive in the liver, the main site of perinatal Treg expansion (4–6), at the interface with gut-derived signals and bacterial metabolites, and central in the regulation of systemic iron metabolism.

Some evidence indicates that, in the *Foxp3*^Cre^ model, the promiscuous Cre activation may result in ectopic recombination of the floxed segment in non-Treg cells (41, 42). However, here we did not observe any sign of off-target CD71 deletion, since Tconvs (Figure 2 and Supplemental Figure 3) and other leucocytes (not shown) highly expressed CD71 in cKO mice. Of note, a recent study (43) has shown that CD71 is crucially required for intestinal Treg expansion in an inducible model of Foxp3-specific *Tfrc* deletion, not affected by promiscuous promoter activation.

In line with published data regarding Tconvs (19, 21), CD71-null Tregs carried less active mitochondria, suggesting a role for impaired homeostasis of these organelles. However, iron can modulate a variety of cellular activities besides mitochondrial metabolism. In CD4^+^ T helper differentiation, iron binds and regulates an RNA-binding protein controlling cytokine production (22). In activated T cells, enzymes involved in deoxyribonucleotide synthesis, DNA replication, and histone and DNA demethylation were among the most critical targets for iron usage, suggesting a pivotal role for iron in epigenetic reprogramming (14). Since the epigenetic landscape (especially DNA demethylation) determines Treg identity (44), it is reasonable to hypothesize that iron influx contributes to Treg expansion also through epigenetic stabilization. Ferritin heavy and light chains are overrepresented in Tregs (45), indicating that iron could be accumulated in ferritin stores. Excessive intracellular iron can become toxic and lead to ferroptosis, a type of cell death characterized by an iron-dependent accumulation of lipid peroxides (46). Of interest, Tregs may be particularly resistant to ferroptosis and oxidative stress, thanks to the activity of antioxidant systems (47, 48). Together, these observations strengthen the idea that Tregs may be particularly efficient in iron absorption and may have developed multiple routes of iron utilization, iron storage, and protection from toxicity.

The lack of expansion of CD71-null Tregs points to a perinatal iron overload response, prompting the hypothesis that perinatal Treg expansion might consume iron and thus prevent iron overload. Accordingly, an early expansion of CD71^+^Ki67^+^ Tregs protected human neonates from a subsequent serum iron increase. Iron scavenging is used by macrophages and neutrophils as a nutritional defense mechanism (17). Other immune cells, i.e., dendritic cells, indirectly control iron availability to gut microbes through the release of hepcidin (49). Our data suggest that perinatal Tregs may exploit iron consumption as a means to favor gut colonization by beneficial bacteria early after birth. Supporting this idea, the hepcidin rise in cKO mice may be secondary to a very early increase in circulating iron. Indeed, iron excess was already compensated at 8–10 days after birth, as testified by the inverse correlation between hepcidin and iron and by the finding of hepatic iron-laden macrophages.

Not only iron abundance but also inflammation can induce hepcidin (16); therefore, we cannot exclude that hepatic Treg deprivation induces an iron overload response indirectly, through the instigation of inflammation. Indeed, antimicrobial molecules released during inflammatory responses interfere with bacterial uptake of several nutrients (including iron) and modulate microbial composition (50). In turn, microbial metabolites can affect intestinal iron absorption through the regulation of HIF-2α activity and ferroportin expression (51). Interestingly, a recent study has shown that a microbial metabolite, pentanoate, assists Tregs in iron uptake and promotes c-Maf^+^ Treg differentiation in the intestine through HIF-2α (43). The key contribution of dysbiosis to the scurfy-like disease is testified by the observation that microbiota manipulation can substantially control inflammation and prolong survival (52). This evidence may point to a model whereby defective Treg expansion in cKO mice induces a cascade of events, which are inflammation, then dysbiosis, then reduced iron absorption and iron overload response. Instead, we propose here the hypothesis that defective expansion and iron consumption by Tregs directly promotes iron overload response, which in turn contributes to gut microbiota modulation, together with inflammation occurring in parallel. We could not formally prove the direct competition for iron between Tregs and bacteria and the existence of a causal link between this event and the scurfy-like disease. Moreover, we did not determine whether iron overload also occurred in liver and colon (sites where CD71 deficiency mostly affected Treg expansion), or whether Tregs represented a preferential iron depot. However, some results point to a minor direct role for inflammation in dictating early microbial and iron dysregulation: i) we could not detect histological signs of prominent immune infiltration in the livers at this age (not shown); ii) hepcidin induction did not correlate with serum IL-6; and iii) we did check for ferroportin (*Slc40a1*) expression in the duodenum of 8- to 10-day-old-mice, but we could not find any difference between cKO mice and controls (not shown).

From an evolutionary viewpoint, Tregs may have evolved in mammals concomitantly to the appearance of lactation, to tolerate bacterial species helpful for milk digestion (53). Therefore, since their origin, Tregs may have engaged in close interactions with bacteria, also mediated by dietary metabolites. Iron is a growth-limiting element for bacteria, and the capacity to scavenge iron from host cells and fluids contributes to their pathogenicity. Beneficial and detrimental bacterial phyla can be differentially sensitive to iron exposure: for instance, dietary iron supplementation decreases Actinobacteria (and less consistently also Bacteroidetes) in the gut (54). Accordingly, species belonging to these 2 phyla were reduced in the fecal microbiota of our cKO mice, presumably in response to iron excess. We have previously reported that activated Tregs expand in neonatal sepsis, inversely correlating with disease severity (55). In mice, the iron-rich peritoneal fluid predisposes neonates to bacterial sepsis (56). It could be speculated that the expansion of CD71^+^ Tregs may be triggered in pathological conditions, such as sepsis, as a means of nutritional immunity.

In conclusion, our data point to a role for iron metabolism in shaping immune regulation, through the CD71-dependent expansion of Tregs. This mechanism may have evolved to counteract pathogenic bacteria in their battle for iron in perinatal life. However, the same mechanism turns from beneficial into detrimental in the adult life, when it is exploited to promote Treg expansion in cancer, a concept known as “antagonistic pleiotropy” (53).

## Methods

### Sex as a biological variable.

As *Foxp3* is X-linked, both male hemizygous and female homozygous mice showed CD71 deletion in the whole Treg population, while only female heterozygous mice displayed Treg mosaicism.

### Human samples.

PB and tumor specimens were obtained from 13 patients with HCC undergoing surgery at Istituto Nazionale dei Tumori “Regina Elena” in Rome. Clinical characteristics are in Supplemental Table 1. Buffy coats from HDs were provided by the Blood Transfusion Centre of Sapienza Università di Roma - Policlinico Umberto I. PBMCs were isolated by density gradient centrifugation through Lympholyte (Cedarlane) and collected in complete RPMI 1640 Dutch-modified medium containing 10% FBS (Gibco), 2 mM l-glutamine (MilliporeSigma), penicillin/streptomycin, nonessential amino acids and sodium pyruvate (EuroClone), and 50 μM β-mercaptoethanol (MilliporeSigma).

Tumor fragments were disrupted on a gentleMACS Octo dissociator (Miltenyi Biotec), in HBSS with Ca^2+^/Mg^2+^ (Gibco), 0.5 mg/mL Collagenase IV (MilliporeSigma), 50 ng/mL DNAse I (Worthington), 6 mg/mL BSA (MilliporeSigma), and 2% FBS (Gibco), prewarmed at 37°C, performing 2 dissociation cycles at room temperature (RT) (program: h_tumor_03) and incubating samples at 37°C, 20 rpm, 10 minutes. Cells were filtered, washed in 40% Percoll (GE Healthcare, now Cytiva), and enriched by Lympholyte.

Whole blood (100 μL) was collected in EDTA-coated microvette tubes (Sarstedt) by heel stick from 53 preterm newborn infants (Supplemental Table 2) at 0–3, 7–10, and 28–31 days after birth. In some neonates, iron concentration was determined in the serum by ELISA at early (8–15 days) and late (28–48 days) time points after birth.

### Mouse models.

*Foxp3*^Cre^ mice (stock 016959) were crossed with *Tfrc*^fl/fl^ mice (stock 028363, both from The Jackson Laboratory). All mice were bred and maintained under conventional conditions at the animal facility of the Dipartimento di Scienze Anatomiche, Istologiche, Medico legali e dell’Apparato locomotore, in Sapienza Università di Roma. The 18.5 c-myc H-Ras V12 p53^–/–^ (“18.5”) HCC line (26), provided by Micol Ravà (European Institute of Oncology, Milan, Italy), was cultured in complete DMEM with high glucose (Gibco) supplemented with 10% FBS (Gibco), 2 mM l-glutamine (MilliporeSigma), penicillin/streptomycin, nonessential amino acids, sodium pyruvate (all from EuroClone), 50 μM β-mercaptoethanol (MilliporeSigma), and 10 mM HEPES (Aurogene). Cells were s.c. injected (10^6^ cells) into the middle flank, and tumor volume (mm^3^) was calculated as (smaller diameter)^2^ × larger diameter.

Liver and tumor samples were mechanically dissociated, and mononuclear cells were enriched through a 40/80 Percoll (GE Healthcare) density gradient, collecting cells at the interface between the 40% and 80% Percoll solutions. Leukocytes were isolated from the colonic LP (57) or VAT (58) according to published protocols. Spleens, lymph nodes, and thymuses were mechanically disrupted on 70 μm filters (Corning), and the obtained cell suspensions were incubated with ammonium-chloride-potassium lysing buffer (Gibco) for 4 minutes at 4°C.

### In vitro assays.

Tregs were magnetically isolated from PB of HDs using the CD4^+^ CD25^+^ CD127^dim/–^ Regulatory T Cell Isolation Kit II (Miltenyi Biotec) and cultured for 14 days with Treg expansion beads (Miltenyi Biotec) and IL-2 (500 IU/mL, Roche). After bead removal, Tregs were labeled with 5 μM CTV (Thermo Fisher Scientific) and cultured 7 days with autologous 10 μM CFSE-labeled Tconvs plus autologous irradiated PBMCs (70 Gy; 4-fold the number of Tconvs), anti-CD3 (1 μg/mL, eBioscience), anti-CD71 blocking antibody (M-A172; BD Biosciences), or isotype control (10 μg/mL). CD4^+^ T cells were magnetically purified from splenocytes of mosaic female mice using CD4^+^ T cell isolation kit (Miltenyi Biotec), labeled with CTV, and cultured 3–4 days with equal numbers of irradiated (35 Gy) splenocytes plus soluble anti-CD3 (1 μg/mL, eBioscience). For survival assay, CD4^+^YFP^+^ Tregs were sorted from splenocytes using an Aurora CS equipped with 405, 488, and 633 nm lasers (Cytek Biosciences), then CTV-labeled and stimulated 3 days with coated anti-CD3 (1 μg/mL) and/or IL-2 (100 IU/mL).

### Flow cytometry.

Cells were first stained with Fixable Viability Dye eFluor780 (eBioscience) for 30 minutes at RT, then surface-stained for 20 minutes at 4°C, and finally intracellular staining was achieved using the Foxp3/Transcription Factor Staining Buffer Set (eBioscience). Before cytokine staining, samples were stimulated 4 hours with Cell Stimulation Cocktail plus protein transport inhibitors (eBioscience) and stained using BD Cytofix/Cytoperm Kit (BD Biosciences). To evaluate transferrin internalization, samples were treated 15 minutes at 37°C with Alexa Fluor 488-Transferrin conjugate (25 μg/mL; Life Technologies). To analyze intracellular labile iron pool, cells were incubated 1 hour at RT with 10 μM Calcein Blue AM (Thermo Fisher Scientific). For the analysis of mitochondria, stainings with MDR, MitoSOX, or TMRM (applying a quenching mode, whereby hyperpolarized cells display decreased fluorescence, ref. 59; as evidenced by the maximal signal obtained with the depolarizing agent FCCP) were performed for 30 minutes at 37°C at 100 nM, 10 μM, and 20 nM, respectively (Thermo Fisher Scientific).

In whole blood samples, after dead cell and surface staining, erythrocytes were lysed with FACS Lysing Solution (BD Biosciences).

A complete list of antibodies is available in Supplemental Table 3.

Data were acquired on an LSR Fortessa (Becton Dickinson) and analyzed with FlowJo software (version 10.7.1; BD Biosciences).

### Immunofluorescence.

Sections from FFPE samples were deparaffinized, rehydrated, and unmasked using Epitope Retrieval Solution, pH 9 (Leica Novocastra) at 98°C for 30 minutes. Subsequently, the sections were brought to RT and washed with PBS. After neutralization of the Fc blocking by 0.4% casein in PBS (Leica Novocastra), sections were incubated with mouse monoclonal CD71 (10F11, 1:80, Leica Novocastra) and rabbit FOXP3 (EPR15038-69, 1:100, Abcam), then with Alexa Fluor 568 goat anti-rabbit (1:300) (Life Technologies) and Alexa Fluor 488 goat anti-mouse. Nuclei were counterstained with DAPI.

### Histopathology.

Lung, liver, ear, and large intestine were washed in PBS; fixed in 10% neutral buffered formalin overnight; washed in water; and paraffin embedded. Formalin-embedded tissues were cut into 4 μm sections and stained with hematoxylin and eosin for histopathological analysis and semiquantitative scoring, performed by 2 expert pathologists, which included the following variables: pulmonary interstitial inflammation and pulmonary interstitial cellularity for lung; periportal and centrilobular inflammatory infiltrate, lobular architectural disarray, sclerosis, and necrosis for liver; epidermal, dermal, and hypodermal inflammatory infiltration, as well as epidermal necrosis and dermal sclerosis, for ear; and cryptitis, chorion inflammatory infiltration and suppression of mucosecretory activity, regenerative/dysplastic alterations, and atrophy for large intestine. All the ordinal variables were scored as severity (0, normal; 1, slight; 2, moderate; 3, marked) and extension (0, absent; 1, focal; 2, multifocal; 3, diffuse). Perls Prussian blue histochemical stain was performed on murine liver sections in accordance with the manufacturer’s protocol (Diapath). Slides were analyzed under a ZEISS Axioscope A1, and microphotographs were collected using a ZEISS Axiocam 503 Color with Zen 2.0 software.

### RNA-Seq analysis.

CD4^+^YFP^+^ Tregs were sorted from splenocytes of mosaic female mice using a FACSAriaIII (BD Biosciences) equipped with 488, 561, and 633 nm lasers and FACSDiva software (BD Biosciences version 6.1.3). To reduce stress, cells were isolated in gentle FACS conditions using a ceramic nozzle of size 100 mm, a low sheath pressure of 19.84 pound-force per square inch (psi) that maintains the sample pressure at 18.96 psi, and an acquisition rate of maximum 1,500 events/s. FACS-sorted cells were more than 95% pure prior to RNA extraction. Total RNA was isolated with Single Cell RNA Purification Kit (Norgen). RNA-Seq library for mRNA sequencing was prepared with SMARTer Stranded Total RNA-Seq Kit - Pico Input Mammalian. All extracted RNA samples were quality-controlled for integrity with a 2100 Bioanalyzer system (Agilent Technologies) and sequenced on a NovaSeq 6000 System (Illumina). Raw data were processed for both format conversion and de-multiplexing by Bcl2Fastq v.2.20 of the Illumina pipeline. Read quality was evaluated using FastQC v.0.11.8 (Babraham Institute) tool. Then adapter sequences were removed with trimgalore v.0.6.6 from FASTQ sequences using the auto detection mode and the following parameters: *--clip_r1 3 --three_prime_clip_r2 3*. Reads were mapped to the mouse Ensembl GRCm38 transcriptome index using salmon (v.1.3.0) (60). Gene-level normalization and differential gene expression analysis were performed with Bioconductor (61, 62) package DESeq2 v.1.28 (63) using the R environment v.4.0. To check for outliers, a regularized log transformation was applied to the count data, and a principal component analysis was generated based on genes showing the highest variance across all samples. The Wald test was used for significance testing, and the resulting FDR *P* values were adjusted for multiple comparisons using the Benjamini and Hochberg method (64). Genes were considered differentially expressed genes (DEGs) only at FDR < 0.05. Gene enrichment analysis was performed by GSEA software comparing data with previously published gene sets.

### Real-time RT-PCR.

Liver fragments (<30 mg) were stored in RNAlater RNA Stabilization Reagent until disruption using gentleMACS M Tubes (Miltenyi Biotec) with Program RNA_01 of gentleMACS Octo Dissociator (Miltenyi Biotec). Total RNA was isolated using RNeasy Kit (QIAGEN), and cDNA was obtained with Superscript VILO cDNA Synthesis Kit (Thermo Fisher Scientific). Quantitative real-time RT-PCR for *Hamp* gene was done in duplicate using the TaqMan Fast Advanced Master Mix and Gene Expression Assay Mm04231240_s1 (Thermo Fisher Scientific) on a StepOne Real-Time PCR System (Applied Biosystems). Sample values were normalized to the Ct value for *Rn18s* gene using the formula 2^−ΔCt^.

### Serum iron and cytokine quantification.

Total iron (Fe^2+^ and Fe^3+^) was measured in serum using iron assay kit (MilliporeSigma). Serum samples at 1:3 dilution were treated with an iron reducer for 30 minutes at RT in the dark, and then a chromogen reacting with the released iron was added. Absorbance was measured at 593 nm with a spectrophotometric multiwell plate reader (Multiskan FC, Thermo Fisher Scientific). Concentration of iron in samples was determined based on the ratio between the amount of iron in unknown sample (nmol) from standard curve and sample volume, and then multiplying for iron atomic mass (55.85 g/mol). For IL-1β and IL-6 determination, sera were diluted 1:4 and analyzed with ELISA kits (Thermo Fisher Scientific and BioLegend, respectively).

### Microbiota analysis.

On average, 14 mg of stools were collected directly from mouse intestine, and microbial DNA was purified using All Prep PowerFecal DNA/RNA Kit (QIAGEN) according to the manufacturer’s protocol. DNA concentration was measured with the NanoDrop 2000 Spectrophotometer (Thermo Fisher Scientific). A defined DNA concentration (5–10 ng/μL) for all samples was used for library preparation. Samples were subjected to robotic (Maxwell RSC Instrument, Promega) PCR amplification, library preparation, and sequencing according to the Illumina 16S metagenomics standardized operational workflow for the 16S rRNA V3-V4 region (16S Metagenomic Sequencing Library Preparation, Part 15044223 Rev. B). Each 16S library was checked for size with an Agilent 2200 Tapestation and quantified with a Qubit 2.0 fluorometer using the Qubit dsDNA HS Assay Kit (catalog Q32851, Thermo Fisher Scientific). Sequencing was performed at the U1052/CNRS 5286 Lyon with an Illumina MiSeq platform, Reagent Kit v3 (catalog MS-102-3003, Illumina), 2 × 300 paired ends.

Raw FASTQ files were analyzed with DADA2 pipeline v.1.14 for quality check and filtering (sequencing errors, denoising, chimeras detection) on a Workstation Fujitsu Celsius R940 (Fujitsu). Filtering parameters were as follows: truncLen=0, minLen=100, maxN=0, maxEE=2, truncQ=11, trimLeft=15. All the other parameters in the DADA2 pipeline for single-end IonTorrent were left as default. Sample coverage was on average more than 99% for all samples, thus meaning a suitable normalization procedure for subsequent analyses. Bioinformatic and statistical analyses on recognized amplicon sequence variants (ASV)were performed with Python v.3.8.2. Each ASV sequence underwent a nucleotide Blast using the NCBI BLAST software (ncbi-blast-2.3.0) and the latest NCBI 16 S Microbial Database (ftp://ftp.ncbi.nlm.nih.gov/blast/db/). Then, the ASVs were merged into species (thus excluding sub-species or strain differences), and a matrix of their relative abundances was built.

Data matrices were normalized and standardized using QuantileTransformer and StandardScaler methods from Sci-Kit learn package v0.20.3. Normalization using the output_distribution = normal option transforms each variable to a Gaussian-shaped distribution, while the standardization results in each normalized variable having a mean of 0 and variance of 1. These 2 steps of normalization followed by standardization ensure the proper comparison of variables with different dynamic ranges, such as bacterial relative abundances or cytokine levels. For microbiota analysis, measurements of α diversity (within-sample diversity), such as richness and Shannon index, were calculated at species level using the SciKit-learn package v.0.4.1. Exploratory analysis of β-diversity (between-sample diversity) was calculated using the Bray-Curtis measure of dissimilarity and represented in principal coordinate analyses (PCoAs), along with methods to compare groups of multivariate sample units (analysis of similarities, ANOSIM; permutational multivariate analysis of variance, PERMANOVA) to assess significance in data point clustering (65). ANOSIM and PERMANOVA were automatically calculated after 999 permutations, as implemented in SciKit-learn package v0.4.1. To visualize a nonsupervised clustering as PCoA, we implemented with custom scripts (Python v3.8.2) a hierarchical clustering analysis with Bray-Curtis metrics and complete linkage method. We implemented PLS-DA and the subsequent VIP as a supervised analysis wherein the VIP values (order of magnitude) are used to identify the most discriminant bacterial species among the cohorts. Bar thickness reports the FR value of the mean relative abundances for each species among the 2 cohorts. Mann-Whitney *U* test and *P* values, without FDR, were used for a fixed sample size as previously described (66), and Kruskal-Wallis tests were used to assess significance for pairwise or multiple comparisons, respectively, considering a *P* < 0.05 as significant.

### Statistics.

The analysis was performed using Prism 9.0 (GraphPad). Data are presented as means ± SD. Mann-Whitney *U* test, or 2-way ANOVA with Tukey’s multiple comparisons test, was used to assess differences between independent experimental groups. Wilcoxon’s matched pairs signed rank test was applied in the analysis of matched samples. Student’s *t* test, 2-tailed and unpaired, was used to analyze in vitro assays with experimental replicates. Spearman’s analysis was used to assess correlations. *P* values less than 0.05 were considered statistically significant.

### Study approval.

Human studies were performed in accordance with the ethical guidelines of the 1975 Declaration of Helsinki and approved by the Sapienza Università di Roma - Azienda Policlinico Umberto I Ethics Committee (Prot. N. 754/18, Prot. N. 5089). Written informed consent was obtained from all patients or from parents.

Mouse experiments were approved by the Italian Ministry of Health, Rome, Italy (authorization no. 1127/2020-PR). All adult mice were killed by cervical dislocation and 8- to 10-day-old mice by decapitation.

### Data availability.

All data generated or analyzed during this study are included in this published article, and raw data are included in the Supporting Data Values file. The RNA-Seq data have been deposited and are available at the following National Center for Biotechnology (NCBI) Gene Expression Omnibus accession code: GSE212688. Raw FASTQ data of 16s sequencing are available at NCBI Sequence Reads Archive - BioProject ID PRJNA1091183.

## Author contributions

SP and IP designed the study and performed data analysis. IP, VC, BB, VL, G Pietropaolo, G Peruzzi, CDC, YA, FG, and VI performed experiments and analyzed results. CT, GS, SC, and VB supervised experiments and provided support. APG, AR, G Tucci, MZ, EP, VP, IR, and IC provided technical and material support. G Terrin, MDC, RP, VDP, and GLG recruited participants and provided samples. SP and IP wrote the first draft of the manuscript. All authors participated in the critical discussion of the results and approved the final paper.

## Supplementary Material

Supplemental data

Supporting data values

## Version 1 07/02/2024

In-Press Preview

## Version 2 08/08/2024

Electronic publication
