# Supplementary material for: Iron capture through CD71 drives perinatal and tumor-associated Treg expansion
Source: JCI Insight. 2024 Jul 2;9(15):e167967. doi: 10.1172/jci.insight.167967 (PMC11383606; doi:10.1172/jci.insight.167967)
Supplement: Supplemental data [file jciinsight-9-167967-s016.pdf]

SUPPLEMENTARY MATERIAL

Table S1

Characteristics of the HCC patients included in the study

Pt	Age (yr)	Sex	Cirrhosis (y/n)	Steatosis (y/n)	HBV (y/n)	HCV (y/n)	AST	ALT	Ferritin (μg/L)	Iron (μg/dL)	Transferrin (mg/dL)
1	61	F	y	n	y	y	72	59	249	n/a	n/a
2	61	M	y	n	n	y	44	37	407	n/a	n/a
3	74	M	y	n	n	n	52	55	34	n/a	n/a
4	84	F	y	n	n	y	50	29	52	n/a	n/a
5	77	F	y	n	n	n	34	15	143	n/a	n/a
6	57	M	y	n	n	y	84	69	277	n/a	n/a
7	80	M	y	n	n	y	86	73	178	n/a	n/a
8	77	M	y	y	n	n	31	27	198,6	85	182
9	75	F	y	n	n	y	52	26	56	15,6	n/a
10	72	M	y	n	n	y	36	21	274	40	225
11	72	M	y	n	n	n	48	35	241	n/a	n/a
12	68	M	y	n	n	n	33	27	9	4,5	n/a
13	64	M	y	y	n	n	44	74	294	116	295

Pt: Patient; yr: years; y/n: yes/no; HBV: Hepatitis B virus; HCV: Hepatitis C virus; AST: aspartate aminotransferase; ALT: alanine aminotransferase; n/a: not available.

Table S2

Baseline clinical characteristics of the neonate and mother populations

**Neonate**, N	53
Pre-natal characteristics	
**GA**, weeks	32.42 ± 2.12
**GA ≤ 28**, weeks (%)	1 (1.89)
**Twins**, N. (%)	36 (67.92)
**Caesarean Section**, N. (%)	50 (94.34)
**Mother's age > 35 years old**, N. (%)	28 (52.83)
**Antenatal antibiotics**, N/available data (%)	12/34 (45.29)
**Antenatal corticosteroids**, N/available data (%)	23/34 (67.64)
**Antenatal iron supplementation**, N/available data (%)	6/26 (23.07)
**Adequate antenatal prophylactic corticosteroids,** N. (%)	36 (67.92)
Post-natal characteristics	
**Female sex,** N. (%)	24 (45.28)
**BW,** N. (%)	1709 ± 444.84
**BW ≤ 1000** grams, N. (%)	4 (33.3)

N: number; GA: gestational age; BW: body weight. Data were expressed as mean ± standard deviation when not specified.

Table S3

List of all the antibodies used in flow cytometry

Marker	Reactivity	Localization	Fluorochrome	Company	Clone
CD127	Human	Surface	PE-Cy7	BioLegend	A019D5
CD14	Human	Surface	APC-eFluor 780	eBioscience	61D3
CD16	Human	Surface	APC-eFluor 780	eBioscience	eBioCB16
CD19	Human	Surface	APC-eFluor 780	eBioscience	HIB19
CD25	Human	Surface	Brilliant Violet 421	BioLegend	mA251
CD3	Human	Surface	Alexa Fluor 488	BioLegend	OKT3
CD4	Human	Surface	Brilliant Violet 510	BioLegend	OKT4
CD45RA	Human	Surface	PE	BioLegend	HI100
CD56	Human	Surface	APC-eFluor 780	eBioscience	CMSSB
CD71	Human	Surface	APC	eBioscience	OKT-9
CD8	Human	Surface	APC-Cy7	BioLegend	RPA-T8
FOXP3	Human	Intracellular	PerCp-Cy5.5	BioLegend	PCH101
Ki67	Human	Intracellular	Alexa Fluor 700	BioLegend	B56
OX40	Human	Surface	Brilliant Violet 605	BioLegend	ACT35
OX40	Human	Surface	PE	BioLegend	ACT35
c-Maf	Mouse	Intracellular	PE-Cy7	eBioscience	sym0F1
CCR8	Mouse	Surface	APC	BioLegend	SA214G2
CD3	Mouse	Surface	BB700	BD Biosciences	145-2C11
CD3	Mouse	Surface	PE-Cy7	BioLegend	17A2
CD4	Mouse	Surface	Brilliant Violet 605	BioLegend	RM4-5
CD4	Mouse	Surface	PerCP-Cy5.5	eBioscience	RM4-5
CD4	Mouse	Surface	Brilliant Violet 786	BD Biosciences	RM4-5
CD44	Mouse	Surface	PE-Cy7	BioLegend	IM7
CD44	Mouse	Surface	Brilliant Violet 510	BD Biosciences	IM7
CD62L	Mouse	Surface	Pe/Dazzle 594	BioLegend	MEL-14
CD71	Mouse	Surface	Brilliant Violet 421	BD Biosciences	C2
CD8	Mouse	Surface	Brilliant Violet 785	BioLegend	53-6.7
DEC1	Mouse	Intracellular	Alexa Fluor 647	Novus Biologicals	Polyclonal
Foxp3	Mouse	Intracellular	APC	eBioscience	FJK-16s
Foxp3	Mouse	Intracellular	PE-eFluor 610	eBioscience	FJK-16s
Helios	Mouse/Human	Intracellular	APC	eBioscience	22F6
IFN-γ	Mouse	Intracellular	Brilliant Violet 421	BioLegend	XMG1.2
IL-13	Mouse	Intracellular	PE	eBioscience	eBio13A
IL-17	Mouse	Intracellular	PerCP-Cy5.5	BioLegend	TC11-18H10.1
IL-4	Mouse	Intracellular	Brilliant Violet 605	BD Biosciences	11B11
IL-5	Mouse	Intracellular	APC	BioLegend	TRFK5
KLRG1	Mouse	Surface	Pe/Dazzle 594	BioLegend	2F1/KLRG1
NFIL3	Mouse	Intracellular	PE	eBioscience	S2M-E19
PD1	Mouse	Surface	PerCP-eFluor710	eBioscience	RMP1-30
Phospho-S6	Mouse	Intracellular	APC	Cell Signaling	D57.2.2E
RORγt	Mouse	Intracellular	PerCP-eFluor 710	Invitrogen	B2D
ST2	Mouse	Surface	Brilliant Violet 421	BD Biosciences	U29-93
TCR-β	Mouse	Surface	Brilliant Violet 510	BD Biosciences	H57-597
TCR-β	Mouse	Surface	Brilliant Violet 711	BioLegend	H57-597

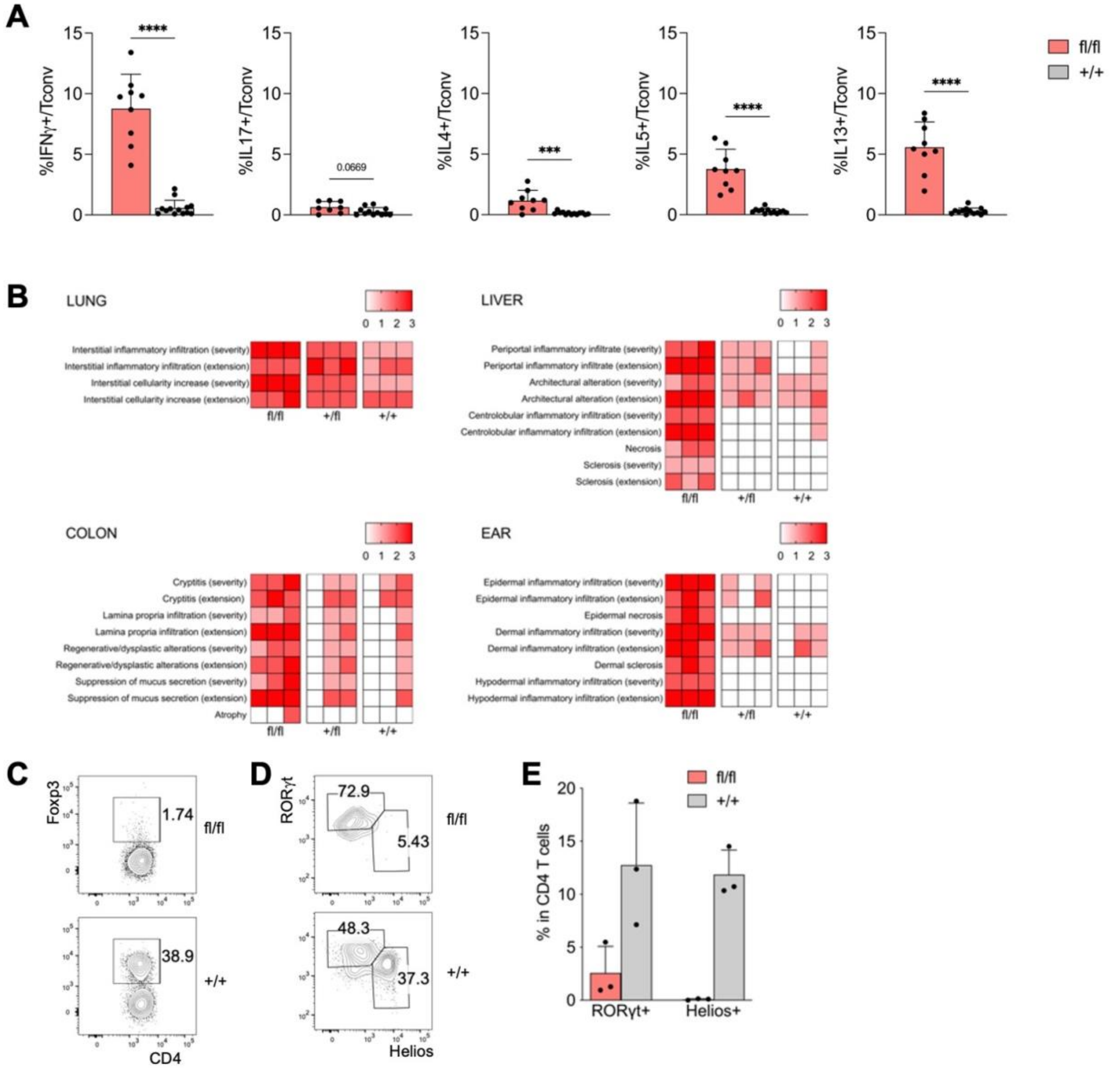

Figure S1. T helper response, histopathological scores and intestinal Tregs in mice with Treg-specific CD71-deficiency

(A) Frequency of cytokine-producing cells in gated Tconvs from the spleens of *Foxp3*$^{Cre/Y}$ *Tfrc*$^{fl/fl}$ mice (fl/fl, n=8-9), compared to *Foxp3*$^{Cre/Y}$ *Tfrc*$^{+/+}$ (+/+, n=12-13) littermates, at 8-10 days of age. *** $P < 0.001$, **** $P < 0.0001$, by Mann-Whitney test. **(B)** Heatmap showing the scores from 0 to 3 assigned to the indicated histopathological features in lung, liver, colon, and ear of *Foxp3*$^{Cre/Y}$ *Tfrc*$^{fl/fl}$ male mice (fl/fl), compared to *Foxp3*$^{Cre/Y}$ *Tfrc*$^{+/fl}$ (+/fl) and *Foxp3*$^{Cre/Y}$ *Tfrc*$^{+/+}$ (+/+) male littermates (3 mice/group), at 3 weeks of age. All the ordinal variables were scored as severity (0, normal; 1, slight; 2, moderate; 3, marked) and extension (0, absent; 1, focal; 2, multifocal; 3, diffuse). **(C-E)** Representative plots of Treg frequency (C) and of RORγt *versus* Helios expression in gated Tregs (D), and cumulative analysis of the percentages of the two Treg subsets in gated CD4 T cells (E), in the colonic LP of *Foxp3*$^{Cre/Y}$ *Tfrc*$^{fl/fl}$ compared to *Tfrc*$^{+/+}$ males (3 mice/group), at 3 weeks of age. Bars indicate means ± SD.

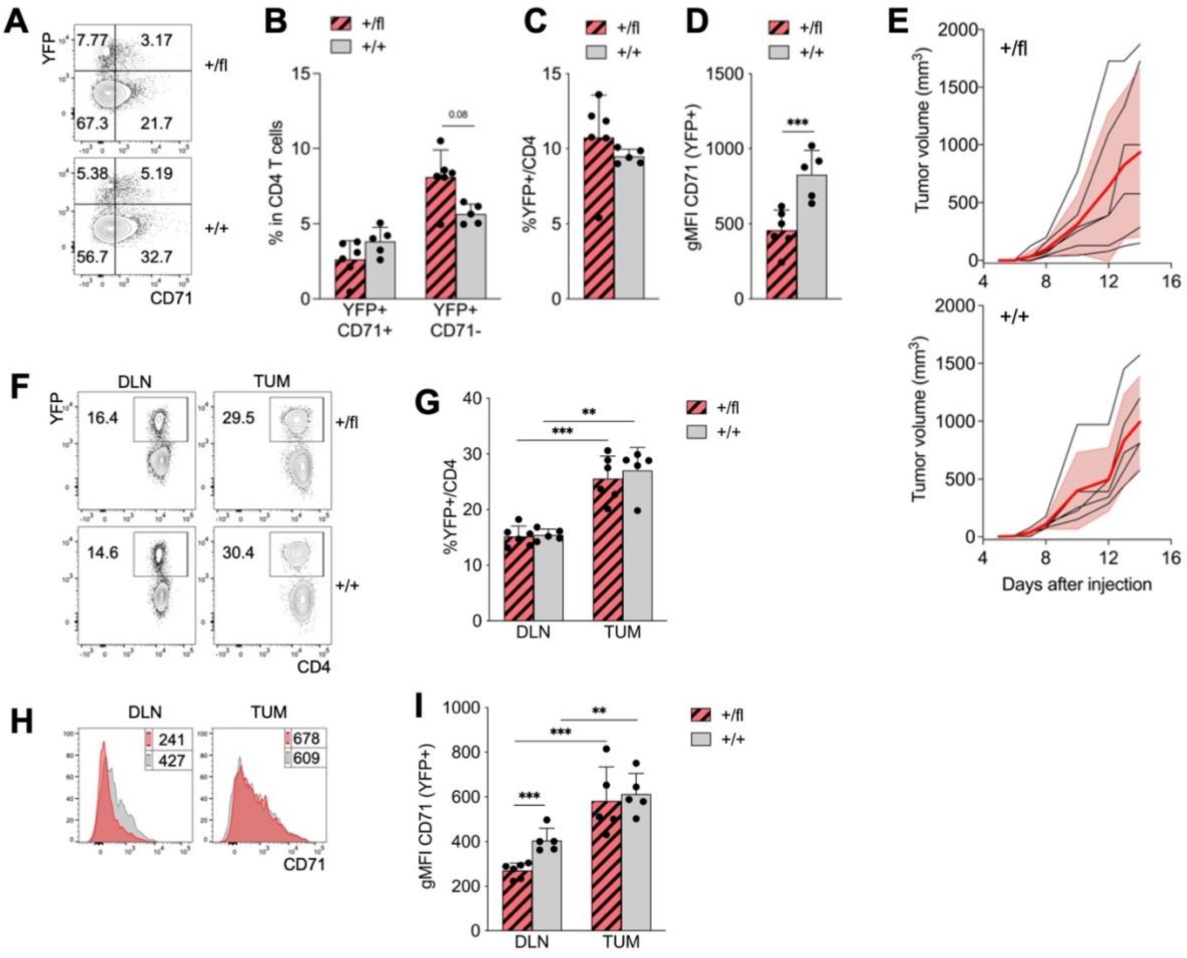

Figure S2. CD71 haploinsufficiency in Tregs is compensated at the tumor site

(A-D) Representative contour plots (A) and cumulative analysis showing the percentages of $CD71^{+}$ and $CD71^{-}$ YFP^{+} cells (B), the percentages of YFP^{+} cells (C), and the expression of CD71 in gated YFP^{+} cells (D), in the spleens of *Foxp3*$^{Cre/Y}$ *Tfrc*$^{+/fl}$ male mice (+/fl), compared to *Foxp3*$^{Cre/Y}$ *Tfrc*$^{+/+}$ (+/+) male littermates (n=5-6 mice/group). Bars indicate means ± SD. *** $P < 0.005$, by Mann-Whitney test. **(E)** Growth curves of the 18.5 HCC cell line, subcutaneously injected in *Foxp3*$^{Cre/Y}$ *Tfrc*$^{+/fl}$ or *Tfrc*$^{+/+}$ male males (n=5-6 mice/group). The red line and red areas represent mean and SD, respectively. **(F-G)** Representative plots (F) and cumulative data (G) of YFP^{+} cell frequency in draining lymph nodes (DLN) and tumors (TUM) of tumor-bearing *Foxp3*$^{Cre/Y}$ *Tfrc*$^{+/fl}$ or *Tfrc*$^{+/+}$ male mice. **(H-I)** Representative plots (H) and cumulative data (I) of CD71 expression in YFP^{+} cells in draining lymph nodes (DLN) and tumors (TUM) of tumor-bearing *Foxp3*$^{Cre/Y}$ *Tfrc*$^{+/fl}$ or *Tfrc*$^{+/+}$ male mice. Numbers indicate the geometric mean fluorescence intensity (gMFI). Bars indicate means ± SD. **$P < 0.01$, *** $P < 0.005$, by Mann-Whitney test.

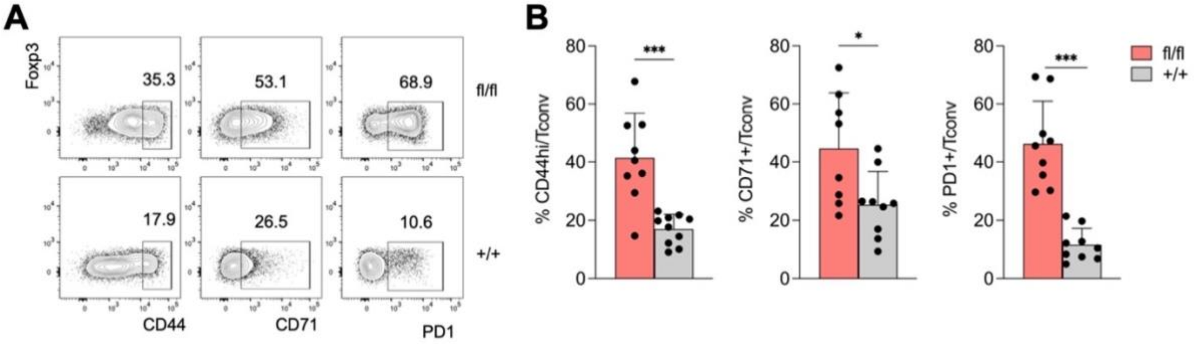

Figure S3. Tconvs are activated in neonates with Treg-specific CD71 depletion

(A) Representative plots and **(B)** cumulative data of the expression of CD44, CD71 and PD1 in gated $CD4^+$ $Foxp3^-$ (Tconvs) from the livers of *Foxp3*$^{Cre/Y}$ *Tfrc*$^{fl/fl}$ male mice (fl/fl, red), compared to *Foxp3*$^{Cre/Y}$ *Tfrc*$^{+/+}$ male littermates (+/+, black), aged 8-10 days. Data are from 9-10 samples per group, pooled from 2 independent experiments. Bars indicate means ± SD. * $P < 0.05$, *** $P < 0.005$, by Mann-Whitney test.

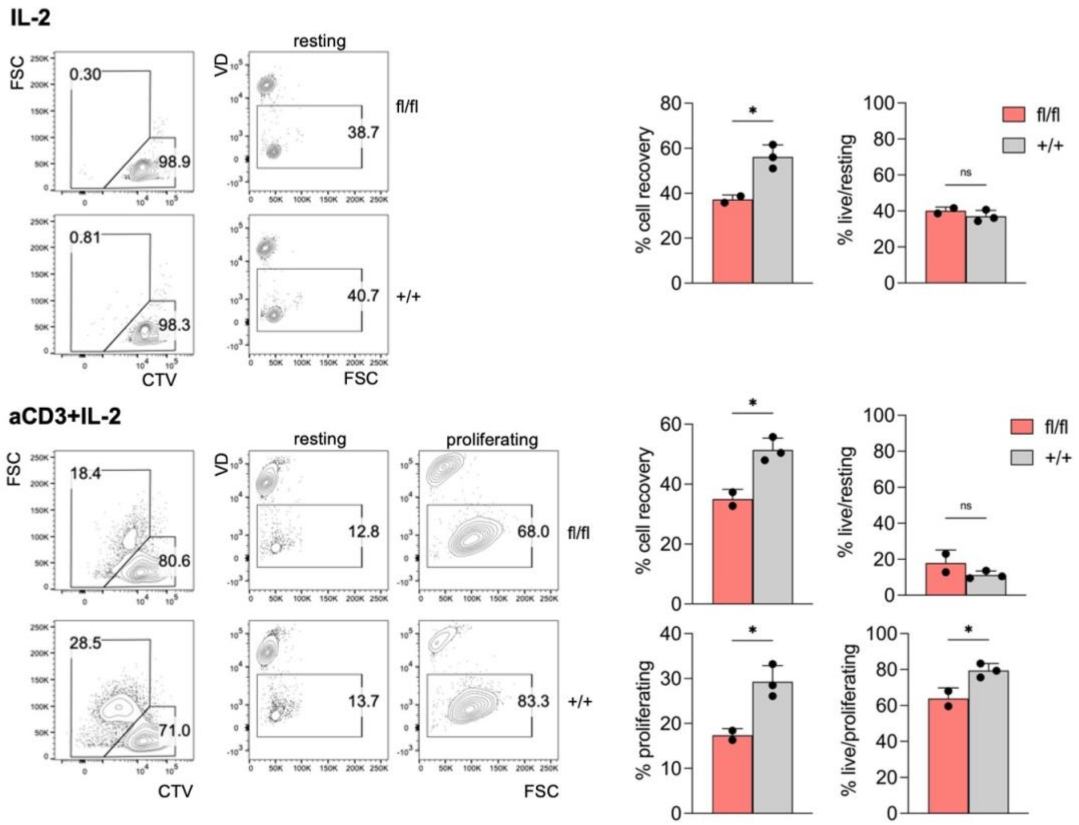

Figure S4. Reduced proliferation and survival in vitro of CD71-deficient Tregs

$CD4^+$ YFP^+ cells were sorted from spleens of *Foxp3*$^{Cre/+}$ *Tfrc*$^{fl/fl}$ female mice (fl/fl) or *Foxp3*$^{Cre/+}$ *Tfrc*$^{+/+}$ female littermates (+/+) of 8-12 weeks of age, labelled with Cell Trace Violet (CTV), and cultured in vitro for 3 days with IL-2 alone or with coated anti-CD3 and IL-2. Then, the percentage of cell recovery was calculated by dividing the number of recovered cells over the number of seeded cells. Cells were analyzed by flow cytometry, using a gating strategy where dead cell were not excluded. Data are from one experiment representative of two. Each condition was tested in 2-3 replicates. * $P < 0.05$, by unpaired Student t test.

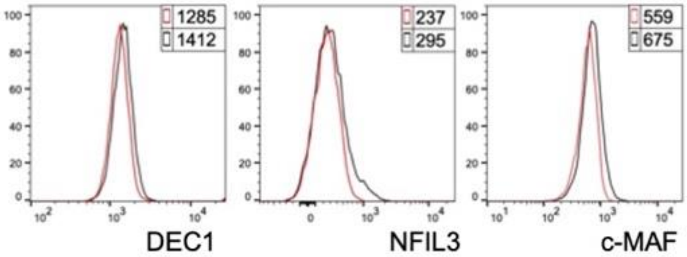

Figure S5. Expression of tissue-Treg markers in CD71-deficient Tregs

Representative histogram overlays showing the intracellular expression of DEC1, NFIL3, ad c-MAF, in $CD4^+$ YFP^+ cells sorted from spleens of *Foxp3*$^{Cre/+}$ *Tfrc*$^{fl/fl}$ female mice (fl/fl, red lines), compared to *Foxp3*$^{Cre/+}$ *Tfrc*$^{+/+}$ female littermates (+/+, black lines), aged 8-12 weeks. Numbers indicate the geometric mean fluorescence intensity (gMFI).

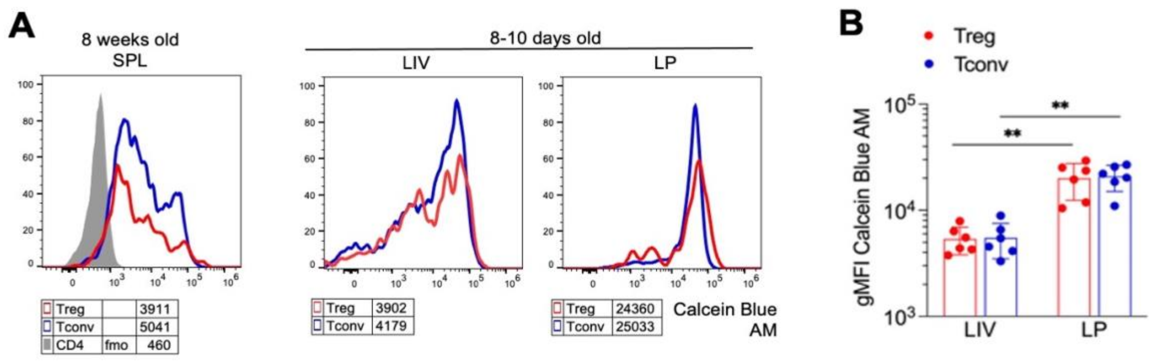

Figure S6. Labile iron pool in Tregs and Tconvs from healthy *Foxp3*^{Cre} mice

(A) Representative histogram overlays of Calcein Blue AM staining in gated CD4^{+}YFP^{+} (Treg) and YFP^{-} (Tconv) from the indicated organs and ages. Numbers indicate the gMFI. **(B)** Cumulative analysis of the gMFI of Calcein Blue AM staining in gated Tregs (red) and Tconvs (blue) in the livers (LIV) or colonic lamina propria (LP) of 8-10 days-old *Foxp3*^{Cre} healthy mice (n=6). ** $P < 0.01$, by Mann-Whitney test.

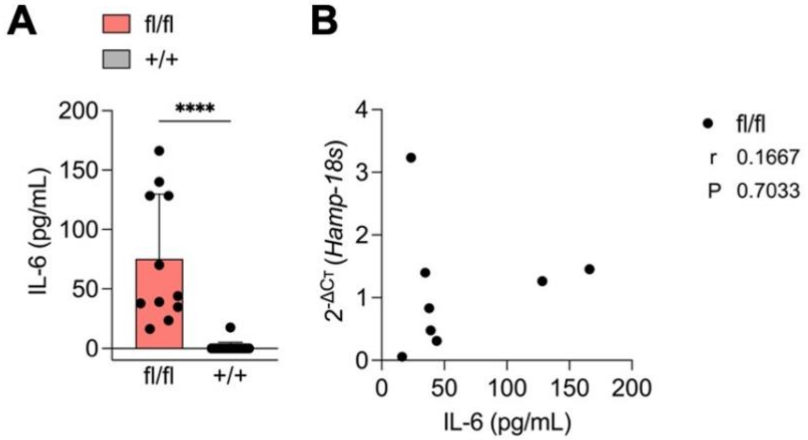

Figure S7. Determination of IL-6 in serum of neonates with Treg-specific CD71 depletion

(A) Cumulative analysis of the concentration of IL-6 determined by ELISA in the serum of *Foxp3*$^{Cre/Y}$ *Tfrc*$^{fl/fl}$ male mice (fl/fl, n=11), compared to *Foxp3*$^{Cre/Y}$ *Tfrc*$^{+/+}$ male littermates (+/+, n=18), aged 8-10 days. Bars indicate means ± SD. **** $P < 0.0001$, by Mann-Whitney test. (**B)** Spearman correlation between serum IL-6 concentrations and hepatic the *Hamp* gene expression in matched samples from *Foxp3*$^{Cre/Y}$ *Tfrc*$^{fl/fl}$ mice (n=8).
